# Vaccine-induced correlate of protection against fatal COVID-19 in older and frail adults during waves of neutralization-resistant variants of concern: an observational study

**DOI:** 10.1016/j.lanepe.2023.100646

**Published:** 2023-05-06

**Authors:** Linnea Vikström, Peter Fjällström, Yong-Dae Gwon, Daniel J. Sheward, Julia Wigren-Byström, Magnus Evander, Oscar Bladh, Micael Widerström, Christian Molnar, Gunlög Rasmussen, Louise Bennet, Mikael Åberg, Jonas Björk, Staffan Tevell, Charlotte Thålin, Kim Blom, Jonas Klingström, Ben Murrell, Clas Ahlm, Johan Normark, Anders F. Johansson, Mattias N.E. Forsell

**Affiliations:** a The Department of Clinical Microbiology, Umeå University, Umeå, Sweden; b The Department of Microbiology, Tumor and Cell Biology, Karolinska Institutet, Solna, Sweden; c The Department of Clinical Sciences, Karolinska Institutet Danderyd Hospital, Stockholm, Sweden; d Familjeläkarna, Stockholm, Sweden; e School of Medical Sciences, Örebro University, Örebro, Sweden; f Department of Clinical Sciences, Clinical Studies Sweden, Forum South, Skåne University Hospital, Lund University, Lund, Sweden; g The Department of Medical Sciences, Clinical Chemistry and SciLifeLab, Uppsala University, Uppsala, Sweden; h The Division of Occupational and Environmental Medicine, Lund University, Lund, Sweden; i Faculty of Medicine and Health, The Department of Infectious Diseases, Karlstad Hospital and Centre for Clinical Research and Education, Region Värmland, Örebro University, Örebro, Sweden; j The Swedish Public Health Agency, Stockholm, Sweden; k The Department of Biomedical Clinical Sciences, Linköpings University, Linköping, Sweden

**Keywords:** COVID-19, Vaccination, Correlate of protection, Vulnerable population, Open cohort study, Longevity of vaccination, Immune monitoring of vulnerable populations

## Abstract

**Background:**

To inform future preventive measures including repeated vaccinations, we have searched for a clinically useful immune correlate of protection against fatal COVID-19 among nursing homes residents.

**Methods:**

We performed repeated capillary blood sampling with analysis of S-binding IgG in an open cohort of nursing home residents in Sweden. We analyzed immunological and registry data from 16 September 2021 to 31 August 2022 with follow-up of deaths to 30 September 2022. The study period included implementation of the 3rd and 4th mRNA monovalent vaccine doses and Omicron virus waves.

**Findings:**

A total of 3012 nursing home residents with median age 86 were enrolled. The 3rd mRNA dose elicited a 99-fold relative increase of S-binding IgG in blood and corresponding increase of neutralizing antibodies. The 4th mRNA vaccine dose boosted levels 3.8-fold. Half-life of S-binding IgG was 72 days. A total 528 residents acquired their first SARS-CoV-2 infection after the 3rd or the 4th vaccine dose and the associated 30-day mortality was 9.1%. We found no indication that levels of vaccine-induced antibodies protected against infection with Omicron VOCs. In contrast, the risk of death was inversely correlated to levels of S-directed IgG below the 20th percentile. The death risk plateaued at population average above the lower 35th percentile of S-binding IgG.

**Interpretation:**

In the absence of neutralizing antibodies that protect from infection, quantification of S-binding IgG post vaccination may be useful to identify the most vulnerable for fatal COVID-19 among the oldest and frailest. This information is of importance for future strategies to protect vulnerable populations against neutralization resistant variants of concern.

**Funding:**

10.13039/501100004359Swedish Research Council, 10.13039/501100004063SciLifeLab via Knut and Alice Wallenberg Foundation, 10.13039/501100001858VINNOVA. Swedish Healthcare Regions, and Erling Persson Foundation.

**Research in context**

**Evidence before this study**

Older individuals in nursing homes are at a high risk of developing severe or fatal COVID-19 after a SARS-CoV-2 infection. Although vaccination has reduced disease burden, vaccine efficacy against infection is reduced due to the emergence of neutralization-resistant variants of concern. However, COVID-19 vaccines remain relatively effective against severe or fatal disease. To better understand correlates of protection in this cohort, we performed a database search on “vaccine-induced correlates of protection” and permutations of this that included “COVID-19” or the term “SARS-CoV-2”. We found that mechanism of protection against fatal disease is not well understood, and it is possible that vaccine-induced antibodies contribute and may predict the risk of fatal disease even after infection with these variants. Identifying a useful correlate of protection could help identify individuals at particular risk and inform future vaccine strategies.

**Added value of this study**

This study outlines an association between levels of vaccine-induced antibodies and 30-day mortality after SARS-CoV-2 infection during waves of neutralization resistant Omicron variants BA.1, BA.2, and BA.5. Such large-scale information with regards to correlates of protection induced by vaccination for vulnerable populations is of value worldwide. Furthermore, the longitudinal study we present comprise 3012 individuals that represent the oldest and most frail population in Sweden, a population that has otherwise eluded scrutiny at a large scale. We also enumerate antibody decline after repeated vaccinations, information that may be useful for determination of future vaccine intervals to provide adequate protection vulnerable groups in the face of new variants of concern.

**Implications of all the available evidence**

Knowledge of the correlates of protection of vaccination is imperative in the design of vaccination programs. This is especially important to strategies to protect vulnerable individuals even in the face of new variants of concern that are unaffected by neutralizing antibodies.

## Introduction

Prior to the rollout of SARS-CoV-2 vaccines, COVID-19 caused substantial morbidity and mortality worldwide, particularly in older individuals. In Sweden, older adults care services are provided in nursing homes staffed and equipped to maintain good quality of life.^1^, ^2^, ^3^ Approximately 80% of the individuals in this population are over the age of 80 and many have comorbidities including dementia.^4^^,^^5^ Early in the pandemic, diagnosed SARS-CoV-2 infection was associated with a 40% excess 30-day mortality among residents at Swedish nursing homes.^5^ Accordingly, nursing home residents were prioritized for vaccination against COVID-19, and 88% had received two vaccine doses as of 1 April 2021.^6^ A majority of these individuals received two doses of BNT162b2 (Pfizer/BioNTech) as their primary vaccination regimen.

A two-dose SARS-CoV-2 mRNA vaccine regimen induces SARS-CoV-2 Spike-directed antibody-responses in the general population and in older individuals,^7^, ^8^, ^9^, ^10^, ^11^ though advanced age has been associated with weaker and less durable responses.^8^^,^^12^^,^^13^ Low titer antibody responses may be even more vulnerable to antibody escape by emerging variants of concern,^14^ raising concerns about the longevity of protection against infection and disease among older nursing home residents. To counteract the waning of specific immune responses, repeated vaccinations have been administered at Swedish nursing homes. A 3rd dose was administered to residents at nursing homes approximately eight months after the primary two-dose regimen. A 4th dose was then recommended to individuals >80-year-old and to all nursing home residents approximately five months after the 3rd dose. For both the third and 4th doses, the vaccine regimens comprised of either BNT162b2 or a half-dose of mRNA-1273 (Moderna). The beneficial effect of these booster doses in reducing infections and mortality in the general population in Sweden has previously been demonstrated.^15^

The efficacy of vaccination with the ancestral stain of S to protect against infection was severely reduced upon the emergence of neutralization resistant Omicron strain,^16^^,^^17^ whereas a high level of protection against hospitalization and fatal COVID-19 remained.^18^ A measurable correlate of protection against COVID-19 mortality would be extremely valuable to identify individuals and cohorts at risk for severe or fatal outcome after a SARS-CoV-2 infection. The aim of the study was to evaluate if levels of vaccine-induced circulating IgG could predict susceptibility for infection or future fatal COVID-19 from infection with variants of concern among nursing home residents. Thus, in a cohort of nursing home residents, we characterized longitudinal Spike (S)-binding antibody responses with high resolution after three or four vaccine-doses and then correlated the results with registry-based data on infections and mortality on an individual basis.

## Methods

### Study design and population

Study subjects living in nursing homes were enrolled if they had received at least one COVID-19 vaccine dose and volunteered to participate in donating capillary blood for immunological analyses and linking with registry data. Informed consent was obtained via an oral and written procedure either the individual or their legal representative (usually next-of-kin) at the nursing home. We used a prospective open cohort design where study subjects could choose to be enrolled every third month, or if already enrolled, continue to donate a capillary blood sample. To enable comparison of immunological responses in nursing home residents, we enrolled community living study subjects under and over the age of 65. Consent procedures, ethical permits and more details on design and population is found in the Supplementary materials. We used the STROBE guidelines for reporting observational studies.^19^

### Data sources, linkage, and missing data

Immunological data from individuals were linked with data in the National vaccine-register (Swedish Public Health Agency), the register containing mandatory reports of all SARS-CoV-2 laboratory and clinical diagnoses in Sweden (SmiNet, Swedish Public Health Agency), and vital status in the total population register (The Swedish Tax Agency) using the person identity number given to all persons registered in Sweden. Register data (all covariates and outcomes) were complete. We performed complete case analyses and sensitivity analyses by replacement of missing data for study subjects missing a S-binding IgG value (a mortality covariate and an immunological outcome). For the Cox proportional hazard regression sensitivity analysis 289/330 missing values fulfilled the criteria of being infection naïve and were used. For capillary samples missing manually registered sampling date, laboratory arrival date we used with subtraction of 3 days for postal service delivery as this was the median time between sampling and arrival for 3567 samples with complete data.

### Sampling strategies

The capillary sampling strategy utilized has been previously described,^20^ with few modifications. Briefly, individual capillary blood sampling kits were sent to study participants via postal services and sampling was conducted by finger pricking by the individual study subject or assisted by an employee at the nursing home.

### S-binding antibody responses, N-binding, and ACE-competition

Circulating IgG against vaccine-derived S proteins were measured in serum or eluted capillary blood as previously described.^21^ Binding antibodies to the N protein of SARS-CoV-2 and antibodies that block ACE-2 binding to the S protein of SARS-CoV-2 Wu-1 were measured using the V-PLEX SARS-CoV-2 Panel 2 (Meso Scale Diagnostics, Maryland, USA) for IgG and ACE-2 according to the manufacturer’s instruction at the SciLifeLab Affinity Proteomics Unit (Uppsala, Sweden). More details on the immunological methods are found in the Supplementary materials.

### *In vitro* neutralization assays

Assessment of the potency of serum to neutralize authentic SARS-CoV-2 *in vitro* was performed under BSL-3 conditions as previously described.^21^ Pseudovirus neutralization was performed as previously described^22^ with modifications as described in Supplementary materials.

### Outcomes

We assessed three outcomes: humoral immune responses to SARS-CoV-2 before and after a vaccine dose, SARS-CoV-2 infection as determined by a SARS-CoV-2 RT-PCR-positive clinical specimen, and SARS-CoV-2–associated mortality as defined by death occurring within 30 days after a SARS-CoV-2-infection. Outcomes were measured from 16 September 2021 to 31 August 2022 with follow-up of deaths until 30 September 2022.

### Data processing

Data processing was carried out by R version 4.1.2 (2021-11-01)—“Bird Hippie”. The code is available at https://github.com/johanssonresearch/Correlate-of-vaccine-protection-against-fatal-COVID-19.

### Covariates

We used the covariate infection-naïve as defined by no previous SARS-CoV-2 infection verified by SARS-CoV-2 PCR-positivity, or by a positive SARS-CoV-2 nucleocapsid antibody measurement (V-plex SARS-CoV-2, Meso Scale Diagnostics). Additional covariates were date of birth, sex, and nursing home caregiver.

### Statistical analyses

Vaccine coverage was calculated based on the vital population at the data for the first administration of the 3rd and 4th mRNA vaccine dose, respectively. We used a cubic spline model with 8 and 10 knots for vaccine dose 3 and 4, respectively, to capture and plot the non-linear trend of S-directed antibody levels in blood over time. We used a generalized additive model with cubic splines to test for difference in antibody levels after dose 3 between the vaccines BNT162b2 and mRNA-1273 and for interaction between the vaccine and the decline in AUC over time with adjustment for age and sex. We used observations of S-binding IgG AUC in infection-naïve study subjects from day 20 to day 80 after dose 3 to approximate the decline by logarithmic regression. SARS-CoV-2-associated 30-day mortality with and without consideration of S-binding IgG antibody levels was analyzed by cumulative Kaplan–Meier estimates and survival at day 30. We used a case-control design with two random infection-naïve controls sampled the same day as each SARS-CoV-2 case as described in the Supplementary materials. For analyses of S-directed antibody level as a correlate of protection against SARS-CoV-2-associated 30-day mortality, we classified the 528 COVID-19 cases into two groups: normal and low antibody responders. Cases with an S-directed antibody AUC lower than given percentile (2.5, 5, 7.5, 10, 12.5, 15, 17.5, 20) at day 60 post vaccine dose 3 were classified as low responders and the other cases as normal responders. We used a Cox proportional hazard model to estimate the risk of death within 30 days adjusting for *covid* (PCR-verified SARS-CoV-2 infection or no infection), *responder* (low or normal AUC-values), *age* (in years), and *sex* (male of female assigned at birth). By inspecting Schoenfeld residuals we observed that the proportional hazards assumption was valid for all variables including the *covid* variable during the first 20 days follow up of deaths. In the interval 20–30 days, however, the assumption of proportionality was violated for the *covid* variable. The limitation was overcome by estimating 95% bootstrap intervals.^23^ We used GraphPad Prism version 9.x for statistical analyses and visualization of comparisons between groups of nursing home residents and community living study subjects. We used non-parametric Mann–Whitney test, Wilcoxon matched-pairs signed rank test for group wise comparisons, or non-parametric Kruskal–Wallis test as appropriate. We used Dunn’s multiple comparison test for post-hoc analyses.

### Role of funding sources

The agencies funding these studies had no role in the study design or interpretation of data.

## Results

### Population and setting

**Fig. 1:**
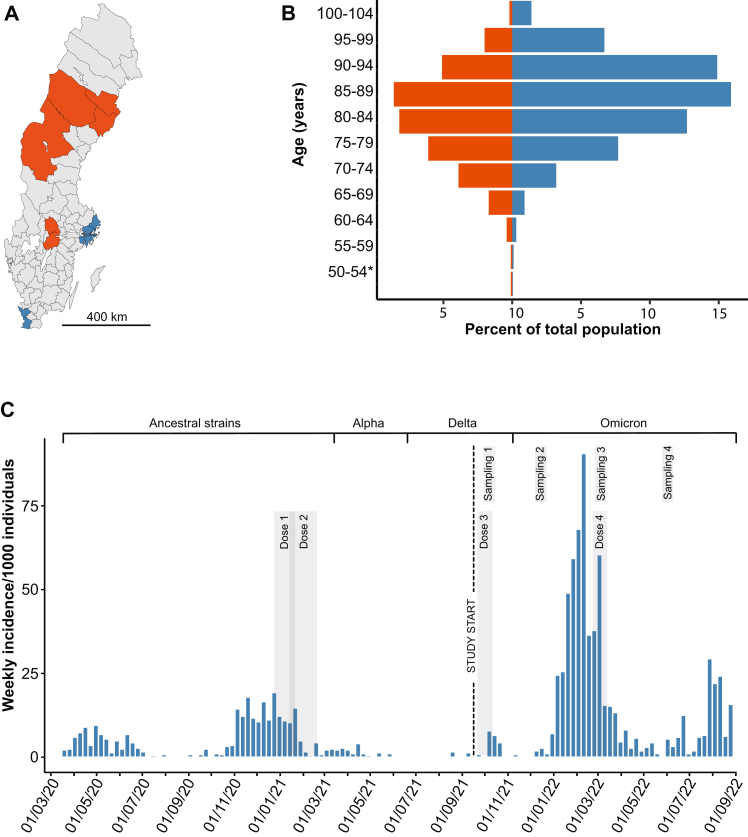
**Location of nursing homes, age and sex characteristics, and the progression of PCR-verified SARS-CoV-2 infections in the study population of 3012 study subjects.** Panel A shows locations in Sweden of nursing homes with recruitment of study subjects in two metropolitan areas (Stockholm and Malmö; blue color) and three less densely populated regions (Västerbotten, Jämtland-Härjedalen, and Örebro; red color). Panel B shows proportions of the total population stratified per age-group of women (blue bars) and men (red bars). An asterisk indicates that one 41-year-old study subject was included. Panel C shows PCR-verified SARS-CoV-2 incidence among the study subjects over the entire pandemic. Timing of vaccine doses, study start, blood sampling periods, dominating virus variant deduced from national genomic surveillance data, periods of vaccination, and blood sampling are indicated. For vaccination and sampling, a gray-shaded interval represents that 80% of the vital cohort was completed.

A total of 3012 nursing home residents from 114 nursing homes were enrolled in an observational open cohort study in Sweden. The enrollment period was from 16 September 2021 to 13 April 2022, starting before the roll-out of a 3rd COVID-19 vaccine dose with a mRNA vaccine. Characteristics of the population at baseline, vaccinations, and capillary sampling for repeated analyses of SARS-CoV-2 antibodies in blood are described in Table 1. Both metropolitan and less densely populated regions were represented (Fig. 1A), and the residents comprised a higher proportion of women than men above 75 years (Fig. 1B). A high weekly incidence within the cohort of PCR-verified SARS-CoV-2 infections with Omicron variants of concern (VOC) facilitated analyses of outcomes with relation to immune responses (Fig. 1C). PCR-verified infections were recorded until 31 August 2022 and deaths until 30 September 2022. At least one S-binding IgG observation in blood was available for 2682 study subjects of which 2631 were immunized fully with a mRNA COVID-19 vaccine. Missing data comprised 330 S-directed IgG values only: 322 subjects donated no blood sample after enrollment and 8 subjects had a failed laboratory analysis. A total of 7071 S-binding IgG observations were available for the fully mRNA-vaccinated cohort.

**Table 1:** Characteristics of the study population.

Characteristic	All regions (N = 3012)	Northern and central regions (N = 2174)	Metropolitan regions (N = 838)
Median age (range)-yr	86 (41–105)	86 (41–105)	85 (59–103)
Female sex-no. (%)	1865 (64.1)	1374 (63.2)	547 (65.3)
History of PCR-verified SARS-CoV-2 at enrollment-no. (%)	405 (13.4)	196 (9.0)	209 (24.9)
**History of vaccination at enrollment-no. (%)**^a^			
1 dose	39 (1.3)	28 (1.3)	11 (1.3)
2 doses	1829 (60.7)	1756 (80.8)	73 (8.7)
3 doses	1144 (38.0)	390 (17.9)	754 (90.0)
**Interval between vaccine doses-days**^a^			
Dose 1–2 median (range)	28 (14–477)	28 (14–477)	21 (17–416)
Dose 2–3 median (range)	245 (57–555)	242 (100–555)	252 (57–532)
Dose 3–4 median (range)	146 (69–353)	143 (69–350)	149 (82–353)
Median enrollment date	08 Oct 2021	05 Oct 2021	22 Nov 2021
**Subjects entering/leaving study-no.**			
16 Sept–27 Nov 2021	2563/160	2047/146	516/14
28 Nov 2021–27 Feb 2022	193/265	71/201	122/64
28 Feb–13 April 2022	256/103	56/81	200/22
14 April–30 Sept 2022	0/313	0/249	0/. 64
**Subjects donating blood sample-no.**			
Sampling period 1: 28 Sept–27 Nov 2021	1570	1570	0
Sampling period 2: 28 Nov 2021–19 Jan 2022	1950	1474	476
Sampling period 3: 28 Feb–05 May 2022	1791	1258	533
Sampling period 4: 19 May–23 July 2022	1647	1218	429
**No. of blood samples donated per person-no.**^b^			
One	540	355	185
Two	654	460	194
Three	798	503	295
Four	690	690	0

a 51 study subjects received ChAdOx1 (Astra-Zeneca) as 1st and/or 2nd dose.

b Including 51 study subjects with ChAdOx1 (Astra-Zeneca) as 1st and/or 2nd dose.

### Robust induction of S-directed IgG after a 3rd and 4th dose of mRNA vaccination among nursing home residents

**Fig. 2:**
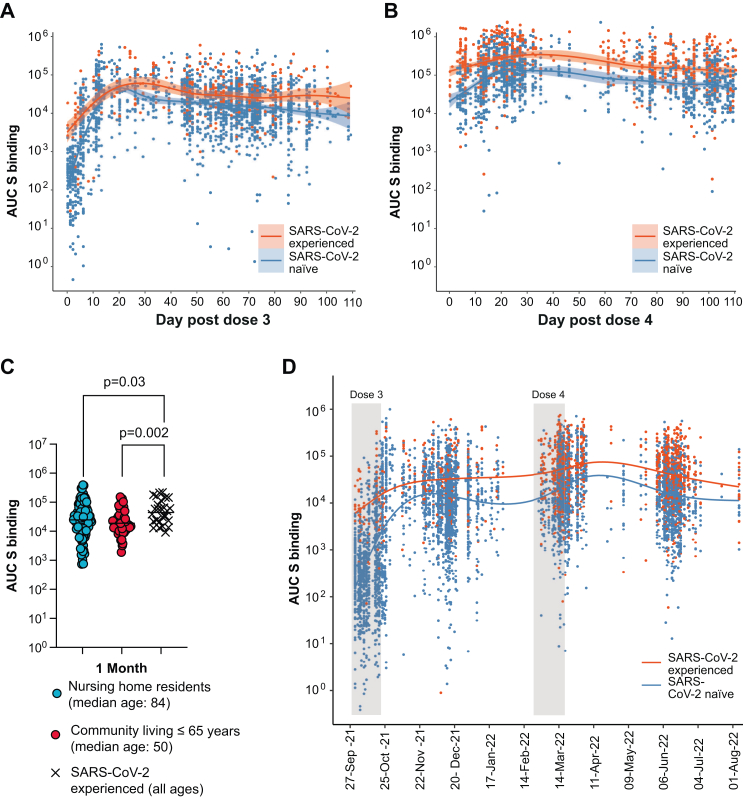
**Dynamics and projection of S-directed IgG responses.** Panel A and B shows S-directed IgG levels in blood with respect to the 3rd and 4th dose of mRNA-vaccination, respectively. Results of repeated capillary blood analyses of previously uninfected (label SARS-CoV-2 infection naïve; blue color) or previously infected (label SARS-CoV-2 experienced; red color) study subjects are shown as dots. Day 0 is the day of vaccination. Shading indicates 95% confidence intervals. Panel C shows comparison of S-directed IgG in the nursing home population (blue) and community living individuals up to the age of 65 (red) and those previously infected with SARS-CoV-2 COVID-19 from both groups (black crosses). Median for each group is shown. Panel D shows S-directed IgG levels obtained by repeated blood sampling at the population level over calendar time. Gray shaded bars represent the interval where the 3rd and 4th vaccination dose, respectively, was completed for 80% of the vital cohort.

The vaccine coverage was 95.9% for the 3rd dose and 80.1% for the 4th dose. We found that >90% of the vaccinees received the 3rd dose within 41 calendar days (median date October 13, 2021), and >90% received the 4th dose within 37 calendar days (median date March 7, 2022). We analyzed S-binding IgG levels before and after the 3rd mRNA vaccination dose with either the standard dose of BNT162b2 or a half-dose mRNA-1273 given after a primary 2-dose regimen of vaccination with BNT162b2 eight months before (Fig. 2A). Previously infected individuals had higher anti-S levels both before and after vaccination. Infection-naïve individuals displayed a 99-fold relative increase (95% CI 74–124) in anti-S IgG levels (median Area Under the Curve (AUC)) 352 (95% CI 316–398) at day-40 to −1 before dose 3 (N = 653); median AUC 34,978 (95% CI 26,718–42,813) at day 14 to day 28 after dose 3 (N = 185). By logarithmic linear regression, we estimated a mean anti-S IgG level decline between day 20 and 80 post dose 3 at 1.3% (95% CI 0.80–1.7) per calendar day, equivalent to a half-life of 70.2 days. The increase in S-binding IgG after dose 3 was reflected in an increased capacity of serum antibodies to outcompete ACE-2 binding to the Wu-1 S-protein *in vitro* (Supplementary materials Figure S1A). The corresponding mean anti-S IgG level decline between day 20 and 80 post dose 4 was 1.5% (95% CI 0.6–2.3), i.e., there was no significant difference in the decline after dose 3 and after dose 4.

By investigating capillary blood samples taken approximately one month after the 3rd dose, we found no difference in S-binding levels between previously uninfected nursing home residents and previously uninfected community living individuals up to the age of 65 (Fig. 2B, Supplementary materials Table S1). For both groups, the levels of S-binding IgG were significantly lower when compared to individuals with a confirmed SARS-CoV-2 infection prior to the 3rd dose.

In comparison with the 3rd dose, the 4th dose led to a more modest 3.8-fold (95% CI 3.2–4.7) relative increase in circulating S-binding IgG (median AUC 14,587 (95% CI 12,383–17,405) from day −40 to −1 before dose 4 (N = 364); median AUC 55,729 (95% CI 48,698–60,079) from day 14–28 post dose 4 (N = 701) (Fig. 2C), again reflected by an increased capacity of serum antibodies to outcompete ACE-2 binding to the Wu-1 S-protein (Supplementary materials Figure S1B). The longitudinal AUC levels for the duration of the study are shown in Fig. 2D.

By generalized additive modeling, we found that median AUC of S-binding IgG after the third mRNA vaccine dose was 1.75 times higher (95% CI 1.39–2.19) with a half dose mRNA-1273 (N = 202) than with BNT162b2 (N = 1576) (Supplementary materials Figure S2). The modeling revealed no significant sex difference in the response levels (ratio 1.01, 95% CI 0.75–1.16). Given the lack of a formal randomization between the vaccine brands in the study design and that our goal was to study overall vaccine responses in the nursing home population, we did not deem that further stratification of data based on vaccine-moiety given was warranted.

### Comparative analysis of vaccine-induced S-specific antibodies after a 4th dose between old and young

**Fig. 3:**
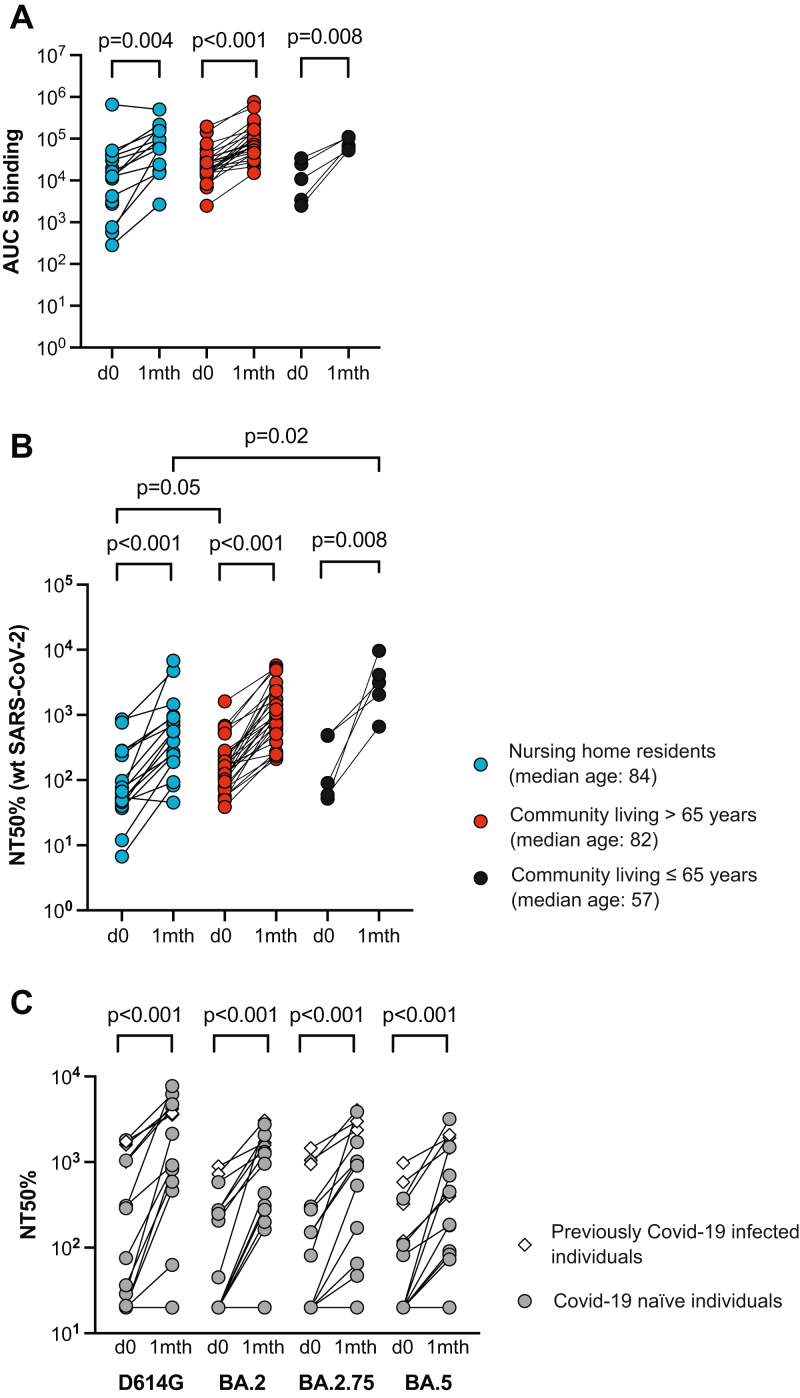
**Detailed effect of booster vaccination.** Samples from nursing home residents (blue circles), community living older individuals (red circles), or community living younger individuals (≤65 years, black circles) were assessed for S-binding IgG (Panel A) or authentic neutralization against Wu-1 isolate of SARS-CoV-2 (Panel B). S-binding is shown as the area under the curve for each sample (AUC) and the reciprocal serum dilution at which 50% neutralization was achieved (NT 50%). Panel C shows neutralization of SARS-Cov-2 S-pseudotyped virus by serum from nursing home residents on day 0 (open circles) and 30 post (black circles) dose 4. Reciprocal serum dilution where 50% neutralization of SARS-CoV-D614G, BA.2, BA.2.75, or BA.5 is shown (NT 50%). Each dot represents one individual.

Venous blood sampling allowed us to do a more detailed serum analysis on days 0, 7–10, and 30 after the 4th vaccine dose. We focused on SARS-CoV-2 unexposed individuals and found that the 4th dose had induced similar increases in S-binding IgG in serum from old persons at nursing homes (median AUC_pre4_ 12,398–AUC_post4_ 66,359), community living persons aged >66 (median AUC_pre4_ 20,885–AUC_post4_ 77,142) or community living persons up to the age of 65 (median AUC_pre4_ 10,843–AUC_post4_ 67,741) (Fig. 3A, Supplementary materials Table S2). This was reflected by significantly increased *in vitro* neutralizing titers against the original SARS-CoV-2 strain among old persons at nursing homes, community living persons over the age of 65 or up to the age of 65 (5.3×, 3.7×, or 6.2× increase, respectively) (Fig. 3B). Statistical analysis demonstrated that samples from 5 individuals under the age of 65 had slightly higher median neutralization titer than samples from 16 nursing home residents after a 4th dose, whereas 27 community-living old individuals had higher neutralization titers than the nursing home residents prior to the 4th dose. We could also demonstrate that the increase in homologous neutralization was associated with enhanced neutralization of Omicron (BA.2, BA.2.75, and BA.5) by serum from nursing home residents (Fig. 3C).

### Infection with relation to booster doses at nursing homes

From study start 16 September 2021 to the end of follow-up of infections 31 August 2022, 25% of the study subjects (765/3012) acquired a primary PCR verified SARS-CoV-2-infection. Twenty-seven study subjects acquired a second PCR verified infection. None was registered with three or more PCR-verified infections during the pandemic. Thirty-two subjects acquired the primary PCR verified infection during the Delta virus dominance from 16 September 2021 to 31 December 2021 and 733 acquired the primary infection during the remainder of the study, where Omicron variants BA.1 and BA.2 were dominant until superseded by BA.5 in June 2022.

We then investigated if vaccine-induced antibody levels had an impact on susceptibility to BA.1 or BA.2 infection. This was done by comparing projected antibody levels among study subjects that acquired PCR-verified infection with the levels among study subjects not acquiring infection. Among 528 previously infection-naïve study subjects vaccinated with three mRNA doses, we found no difference in levels of S-specific IgG to study subjects that subsequently acquired infection over the corresponding 14-day periods after the 3rd mRNA vaccine dose (Supplementary materials Figure S3).

### Antibody levels in relation to mortality at nursing homes

Until the end of follow-up of deaths, 841 study subjects died: 243 from 16 September to 31 December 2021, and 598 from 1 January to 30 September 2022. From study start to end of register follow-up 30 September 2022, 65 deaths occurred within 30 days after a PCR-verified SARS-CoV-2 infection, five during Delta and 60 during the Omicron waves. We observed that short life expectancy after enrollment was a major reason for missing S-directed IgG: median time to 168 deaths among 330 subjects missing all IgG values was 32 days while it was 121.5 days for 613 deaths among 2682 subjects with at least one IgG value.

**Fig. 4:**
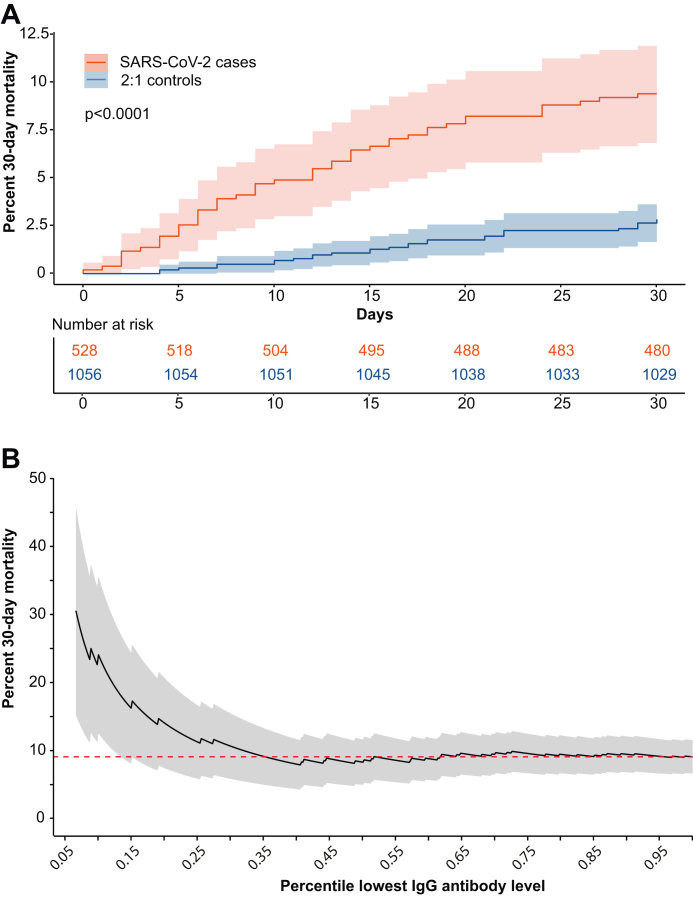
**SARS-CoV-2-associated 30-day mortality.** Panel A shows cumulative 30-day SARS-CoV-2-associated mortality among 528 previously infection naïve nursing home residents that were vaccinated with three or four mRNA vaccine doses before the infection (red). Control study subjects (blue) were sampled 2:1 adjusted to calendar time. Panel B shows the SARS-CoV-2 related 30-day mortality with respect to S-binding IgG levels in the nursing home population. The mortality is shown in relation to the percentile range of lowest antibody levels among the 528 SARS-CoV-2 cases and refers to antibody levels at day 60 post vaccine dose 3.

Among the 528 SARS-CoV-2 case subjects that acquired their first SARS-CoV-2 infection after the 3rd or the 4th vaccine dose, and where an appropriately timed analysis of S-directed IgG was available, the 30-day mortality was 9.1% (48 deaths/528 cases). In comparison, the 30-day mortality was 2.6% (27/1056) among control subjects sampled at the same day (Fig. 4A). We found no difference in 30-day mortality between individuals that acquired infection after the 3rd (32 deaths/362 cases) or the 4th mRNA vaccine dose (16 deaths/166 cases; Kaplan–Meier logrank test, 95% CI 0.59–2.04) (Supplementary materials Figure S4).

The 30-day mortality was higher in subjects with low levels of S-directed IgG among the 528 SARS-CoV-2 cases and this effect plateaued to reach the population average at approximately the 35th percentile (Fig. 4B). Sensitivity analyses using stepwise Kaplan–Meier curves showed that an increased 30-day mortality effect was significant below to the 20th percentile of circulatory S-binding IgG (Supplementary materials Figure S5). For example, subjects with the 10th percentile lowest S-binding IgG (N = 53) had projected AUCs at <479 at day 60 post the 3rd mRNA vaccine dose and 24.5% (13/53) 30-day mortality after PCR-verified SARS-CoV-2 infection. This contrasted with 7.4% (35/475) mortality among cases with a measured AUC ≥479 (N = 475). We further modeled 30-day mortality using Cox proportional hazards regression to take additional risk factors for fatal SARS-CoV-2 into account. By this analysis, we found that nursing home residents with an AUC <479 were at increased risk (hazard ratio 3.61, 95% CI 1.92–6.90) as were residents infected with SARS-CoV-2 (hazard ratio 2.70, 95% CI 1.43–4.90) or those of male sex (hazard ratio 2.19, 95% CI 1.85–5.85). Finally, and as well established, we found that each one-year increase in age increased the risk for SARS-CoV-2-associated mortality (hazard ratio 1.04, 95% CI 1.01–1.08) (Supplementary materials Table S3). We found no significant interaction effect between an AUC <479 and increased risk to acquire a SARS-CoV-2 infection (95% CI 0.92–1.81). To investigate effects of missing data in the complete case analysis, we performed imputation and found that S-directed IgG AUC <479 remained significant both with random and non-random missing data replacement (Supplementary materials Table S3). Taken together, the Cox modeling suggests that on top of an increased mortality imposed by SARS-CoV-2 infection there is an overall increased 30-day mortality among individuals with low S-antibody responses.

## Discussion

A nationally coherent vaccination scheme and a strong clinical staff network at nursing homes and a proven strategy to collect capillary blood samples^20^ allowed us to prospectively analyze vaccine-induced humoral immunity and to combine these data with high-quality register data on the individual level. The nursing home population in Sweden was continuously surveilled by rigorous PCR-based diagnostics of suspected SARS-CoV-2 infections during the study, in accordance with national and regional recommendations. This facilitated meaningful follow-up of 30-day mortality from SARS-CoV-2 infection and a comparison with vaccine-induced antibody responses.

By a detailed analysis of immune responses, we verified an increase in cross-neutralizing antibodies against Omicron lineages BA.1, BA.2.75, and BA.5 after the 4th dose of mRNA vaccination. This is consistent with increased antibody-mediated neutralization of the homologous original SARS-CoV-2 strain to increase protection also against *in vitro* infection with VOCs.^14^^,^^24^ The relatively lower boost effect of the 4th vaccination, as compared with the 3rd may be explained, at least in part, by a previously described feedback mechanism where pre-existing specific antibodies can limit re-activation of humoral immunity.^25^, ^26^, ^27^ Our analysis demonstrated that both the 3rd and the 4th vaccine dose induced a boost effect on SARS-CoV-2 S-binding that was similar between the study cohort and comparator groups of community living individuals. We also found that the 4th vaccine dose induced significantly higher neutralizing antibody titers in sera from five younger community living individuals than in sera from nursing home residents, but not from community living individuals over the age of 65. These data support that age related variability of the immune response to two doses of mRNA vaccination had remained even after additional booster doses.^8^^,^^12^

The study period included the first omicron waves in Sweden and started at a time point when the 3rd mRNA vaccination was distributed to nursing home residents. We found no pattern supporting that higher levels of circulating S-binding IgG protected against SARS-CoV-2 infection. This may be attributable to the described significant escape from neutralizing antibodies by Omicron.^16^^,^^28^ It has previously been shown that vaccine-induced neutralizing antibodies provided a correlate of protection against infection with the ancestral strain or relatively homologous VOCs.^29^^,^^30^ Our results complement these data and demonstrates that S-binding IgG antibody levels provide a correlate of protection against fatality related to infections with VOCs.

Importantly, our analyses identify a threshold titer of anti-S IgG in capillary blood taken after the 3rd vaccine dose below which there was significantly increased risk of fatal COVID-19. We found that the study subjects having the 20th lowest percentile of S-binding IgG 60 days after the 3rd vaccine dose had a significantly increased risk of fatal COVID-19. The risk effect included study subjects that in addition to the 3rd vaccine dose also received the 4th dose. We conclude that S-specific IgG against the original SARS-CoV-2 strain can serve as a correlate of protection against fatal COVID-19 after infection with SARS-CoV-2 variants that demonstrate significant immune escape from neutralizing antibodies. An additional interesting observation was that SARS-CoV-2-associated fatalities occurred within 20 days of a confirmed SARS-CoV-2 infection in all individuals with low S-specific IgG in blood. This could indicate a role of non-neutralizing antibodies to control the infection, as previously suggested.^31^ However, it is also possible that low levels of S-IgG indicate an overall impairment of the adaptive immune system to mount broad antiviral B- and T cell responses, which could further explain why these individuals are particularly vulnerable. Importantly, individuals with low S-specific IgG in circulation represented a minority, and our data demonstrate that between 70 and 80% of all individuals at Swedish nursing homes have achieved maximal vaccine-induced protection against fatal COVID-19 after 3 or 4 doses of monovalent mRNA vaccination.

The study has certain limitations; Nursing home residents at a terminal stage of life were not enrolled in the study, we lacked detailed information comorbidities and data to describe severity of infection was not accessible. Hence, this study was limited to an understanding of diagnosed SARS-CoV-2 infections and 30-day mortality. Moreover, there were missing immunological data for some study subjects, we could not perform neutralization assays on the small sample volumes acquired from capillary blood, and we lacked information on mucosal Spike-specific IgA levels that may be importance to for protection against infection.^32^^,^^33^ These limitations highlight the need for further studies to address these issues and provide a more comprehensive understanding of the impact of vaccination and correlates of protection against SARS-CoV-2 variants of concern.

In conclusion, we have implemented a national surveillance platform to monitor immunity, infections, and mortality within a cohort of nursing home residents. By this, we identified a vaccine-induced threshold level of S-directed IgG in blood that defined a subpopulation of residents that was highly vulnerable to fatal COVID-19. Below the threshold, IgG levels were inversely correlated with the risk to die within 20 days if a verified infection with neutralization resistant VOCs. Despite the continuous emergence of new variants escaping antibody-mediated neutralization, the knowledge of an IgG threshold level defining increased risk to die from an infection and the knowledge of antibody-half life should make it feasible to predict long term protective effect after vaccination. The data presented here should be valuable in COVID-19 vaccine policy decisions on the timing of booster doses for the oldest and frailest.

## Contributors

AFJ and MNEF conceived and designed the study. CA, JK, MNEF, JN, JK, BM, and AFJ obtained research funding. PF, JB, and AFJ developed the statistical analysis plan. LV, PF, AFJ, and MNEF did the formal statistical analysis. MW, CM, LB, GR, OB, ST, CT, CA, JN, AFJ, and JWB were responsible for study subject enrollment. LV, YDG, DJS, JWB, ME, MÅ, KB, JK, and BM performed experiments and analyzed data. LV, PF, AFJ, and MNEF had full access to all the data reported in the study. MNEF and AFJ wrote the first draft of the manuscript and all authors revised and edited the paper. AFJ and MNEF shared the final responsibility for the decision to submit for publication.

## Data sharing statement

Data sharing is regulated by the General Data Protection Regulation 2016/679, the Swedish Law on Biobanking and approved ethical permits. The data material in this study comprises personally identifiable and sensitive information that cannot be shared.

## Declaration of interests

All authors declare no competing interests.

## References

[1] COVID-19 Forecasting Team (2022). Variation in the COVID-19 infection-fatality ratio by age, time, and geography during the pre-vaccine era: a systematic analysis. Lancet.

[2] Statens offentliga utredningar SR (2020). https://coronakommissionen.com/wp-content/uploads/2020/12/sou_2020_80_aldreomsorgen-under-pandemin_webb.pdf.

[3] Statens offentliga utredningar SR (2020). https://www.government.se/4af26a/contentassets/2b394e1186714875bf29991b4552b374/summary-of-sou-2020_80-elderly-care-during-the-pandemic.pdf.

[4] Bergman J., Ballin M., Nordström A., Nordström P. (2021). Risk factors for COVID-19 diagnosis, hospitalization, and subsequent all-cause mortality in Sweden: a nationwide study. Eur J Epidemiol.

[5] Ballin M., Bergman J., Kivipelto M., Nordström A., Nordström P. (2021). Excess mortality after COVID-19 in Swedish long-term care facilities. J Am Med Dir Assoc.

[6] Folkhälsomyndigheten (2021). https://share.mediaflow.com/se/?SDFE9O7K3O.

[7] Anderson E.J., Rouphael N.G., Widge A.T. (2020). Safety and immunogenicity of SARS-CoV-2 mRNA-1273 vaccine in older adults. N Engl J Med.

[8] Collier D.A., Ferreira I., Kotagiri P. (2021). Age-related immune response heterogeneity to SARS-CoV-2 vaccine BNT162b2. Nature.

[9] Baden L.R., El Sahly H.M., Essink B. (2021). Efficacy and safety of the mRNA-1273 SARS-CoV-2 vaccine. N Engl J Med.

[10] Polack F.P., Thomas S.J., Kitchin N. (2020). Safety and efficacy of the BNT162b2 mRNA Covid-19 vaccine. N Engl J Med.

[11] Walsh E.E., Frenck R.W., Falsey A.R. (2020). Safety and immunogenicity of two RNA-based Covid-19 vaccine candidates. N Engl J Med.

[12] Tober-Lau P., Schwarz T., Vanshylla K. (2021). Long-term immunogenicity of BNT162b2 vaccination in older people and younger health-care workers. Lancet Respir Med.

[13] Schwarz T., Tober-Lau P., Hillus D. (2021). Delayed antibody and T-cell response to BNT162b2 vaccination in the elderly, Germany. Emerg Infect Dis.

[14] Pajon R., Doria-Rose N.A., Shen X. (2022). SARS-CoV-2 Omicron variant neutralization after mRNA-1273 booster vaccination. N Engl J Med.

[15] Nordström P., Ballin M., Nordström A. (2022). Effectiveness of a fourth dose of mRNA COVID-19 vaccine against all-cause mortality in long-term care facility residents and in the oldest old: a nationwide, retrospective cohort study in Sweden. Lancet Reg Health Eur.

[16] Andrews N., Stowe J., Kirsebom F. (2022). Covid-19 vaccine effectiveness against the Omicron (B.1.1.529) variant. N Engl J Med.

[17] Planas D., Saunders N., Maes P. (2022). Considerable escape of SARS-CoV-2 Omicron to antibody neutralization. Nature.

[18] Abu-Raddad L.J., Chemaitelly H., Ayoub H.H. (2022). Effect of mRNA vaccine boosters against SARS-CoV-2 Omicron infection in Qatar. N Engl J Med.

[19] Vandenbroucke J.P., von Elm E., Altman D.G. (2007). Strengthening the reporting of observational studies in epidemiology (STROBE): explanation and elaboration. PLoS Med.

[20] Byström J.W., Vikström L., Rosendal E. (2023). At-home sampling to meet geographical challenges for serological assessment of SARS-CoV-2 exposure in a rural region of northern Sweden, March to May 2021: a retrospective cohort studySerological assessment of SARS-CoV-2 exposure in northern Sweden by the use of at-home sampling to meet geographical challenges in rural regions. Euro Surveill..

[21] Normark J., Vikström L., Gwon Y.D. (2021). Heterologous ChAdOx1 nCoV-19 and mRNA-1273 vaccination. N Engl J Med.

[22] Sheward D.J., Kim C., Fischbach J. (2022). Evasion of neutralising antibodies by omicron sublineage BA.2.75. Lancet Infect Dis.

[23] Stensrud M.J., Hernan M.A. (2020). Why test for proportional hazards?. JAMA.

[24] Qu P., Faraone J., Evans J.P. (2022). Neutralization of the SARS-CoV-2 Omicron BA.4/5 and BA.2.12.1 subvariants. N Engl J Med.

[25] Forsell M.N.E., Kvastad L., Sedimbi S.K., Andersson J., Karlsson M.C.I. (2017). Regulation of subunit-specific germinal center B cell responses to the HIV-1 envelope glycoproteins by antibody-mediated feedback. Front Immunol.

[26] Zhang Y., Meyer-Hermann M., George L.A. (2013). Germinal center B cells govern their own fate via antibody feedback. J Exp Med.

[27] Tas J.M.J., Koo J.H., Lin Y.C. (2022). Antibodies from primary humoral responses modulate the recruitment of naive B cells during secondary responses. Immunity.

[28] Willett B.J., Grove J., MacLean O.A. (2022). Publisher correction: SARS-CoV-2 Omicron is an immune escape variant with an altered cell entry pathway. Nat Microbiol.

[29] Gilbert P.B., Donis R.O., Koup R.A., Fong Y., Plotkin S.A., Follmann D. (2022). A Covid-19 milestone attained–a correlate of protection for vaccines. N Engl J Med.

[30] Gilbert P.B., Montefiori D.C., McDermott A.B. (2022). Immune correlates analysis of the mRNA-1273 COVID-19 vaccine efficacy clinical trial. Science.

[31] Zhang A., Stacey H.D., D'Agostino M.R., Tugg Y., Marzok A., Miller M.S. (2022). Beyond neutralization: Fc-dependent antibody effector functions in SARS-CoV-2 infection. Nat Rev Immunol.

[32] Havervall S., Marking U., Svensson J. (2022). Anti-spike mucosal IgA protection against SARS-CoV-2 Omicron infection. N Engl J Med.

[33] Marking U., Bladh O., Havervall S. (2023). 7-month duration of SARS-CoV-2 mucosal immunoglobulin-A responses and protection. Lancet Infect Dis.

